# Supplementary material for: Assessment of Blood Endothelial Cell Biomarkers in Women and Men with Abnormal Body Mass and Paroxysmal Atrial Fibrillation Based on CHA2DS2-VASC Score: A Retrospective Study
Source: Int J Mol Sci. 2025 Apr 11;26(8):3627. doi: 10.3390/ijms26083627 (PMC12027103; doi:10.3390/ijms26083627)
Supplement: Supplementary file 1 [file ijms-26-03627-s001.zip › ijms-3528321-supplementary.pdf]

Table S1. Characteristics of females with paroxysmal AF according to low (<3) and high (≥3) CHA2DS2-VASc score. The unpaired data were analyzed using parametric (unpaired t-test) and nonparametric (Mann-Whitney) t-tests. Categorized data were analyzed with the Chi-square test. A p-value < 0.05 was considered significant.

Abbreviation: BMI - body mass index; EHRA - European Heart Rhythm Association; CHA2DS2-VASc - Congestive heart failure, Hypertension, Age ≥ 75, Diabetes, Stroke, Vascular disease, Age 65–74, Sex (female); HAS-BLED - Hypertension, Abnormal renal/liver function, Stroke, Bleeding history or predisposition, Labile INR, Elderly (>65 years), Drugs/alcohol concomitantly; EF – ejection fraction; TnT - cardiac troponin T; CK-MB – creatine kinase MB, specific for the heart muscle; SBP - systolic blood pressure; DBP- diastolic blood pressure; ST-2 – suppression of tumorigenicity 2 (potential biomarker associated with cardiac stress and fibrosis); TSH – thyroid stimulating hormone; GFR, glomerular filtration rate; sCD40L – soluble ligand CD40 (release from activated platelets); t-PA – tissue plasminogen activator; PAI-1 – plasminogen activator inhibitor 1; sVCAM-1 – soluble vascular cell adhesion molecule 1; sICAM-1- soluble intercellular adhesion molecule; vWF - von Willebrand

factor; sTM – soluble thrombomodulin; ACE - angiotensin-converting enzyme inhibitor; VKA, vitamin K antagonist; NOAC, non-vitamin K antagonist oral anticoagulants; ARB - angiotensin II receptor blocker

**Parameters**	**Female CHA2DS2-VASC <3 n=25**	**Female CHA2DS2-VASC ≥3 n=22**	**p**
Age, years	62 (43-73)	68 (60-73)	<0.0001
BMI, kg/m^2	30.9 (23.3-42.5)	32.3 (25.3-36.7)	0.8451
BMI>25≤45	23 (92)	22 (100)	0.1752
Il-6, pg/mL	3.2 (2.2-11.4)	3.4 (2.0-9.2)	0.6080
EHRA 1	0	0	-------
EHRA 2	15(60)	9(41)	0.1914
EHRA 3	10(40)	10 (45)	0.7059
EHRA 4	0	3(14)	0.0564
Mean HAS-BLED	1 (0-2)	2 (1-3)	<0.0001

% EF	59 (50-60)	59 (50-61)	0.8972
TnT, µg/L	0.007 (0.003-0.084)	0.007 (0.003-0.87)	0.4097
CK-MB, U/L	15 (10-37)	15 (11-27)	0.8198
SBP	125 (100-160)	130 (110-160)	0.0884
DBP	75 (52-91)	80 (60-95)	0.2715
D-Dimers, mg/dl	0.19 (0.03-1.36)	0.16 (0.07-0.54)	0.6430
ST2	1.2 (0.50-5.0)	0.78 (0.51-3.6)	0.6157
TSH, µU/mL	2.5 (0.66-7.7)	1.2 (0.40-6.3)	0.0037
GFR, mL/min	67 (44-114)	64 (40-91)	0.0574
sCD40L, pg/mL	559 (261-985)	512 (179-845)	0.1118
Cholesterol, mg/dL	198 (160-267)	177 (132-290)	0.2159
Glucose, mg/dL	98 (88-127)	100 (87-112)	0.6504
Smoking, n(%)	2(8)	1 (5)	0.6288
Endothelial cell markers			
t-PA, ng/mL	1,5 (0.67-3.1)	1.5 (0.69-1.9)	0.9621
PAI-1, ng/mL	18.0 (12.1-32.5)	17.8 (10.4-37.5)	0.7554
sVCAM-1, ng/mL	300.4 (111.4-601.3)	204.6 (70.1-590.9)	0.0560
sICAM-1, ng/mL	36.1 (16.7-261.9)	34.3 (19.3-295.9)	0.4244
vWF, ng/mL	1.8 (1.2-3.2)	1.9 (1.6-4.7)	0.2284
sTM, ng/mL	3.6 (2.4-5.1)	3.6 (2.3-5.7)	0.5866
Comorbidities and medications			
Dyslipidemia, n(%)	15 (60)	8 (36)	0.1058
Hypertension, n(%)	14 (56)	19 (84)	0.0231
Heart Failure, n(%)	1(4)	0(0)	0.343
Thyroid Diseases, n(%)	11 (44)	6 (27)	0.2337
Statins, n(%)	10(40)	12(54)	0.3187
ACE inhibitor, n (%)	5(20)	5 (23)	0.8197
VKA, n(%)	7(28)	10 (45)	0.214
NOAC, n(%)	18 (72)	12(55)	0.214
Beta-blockers, n(%)	23(92)	19 (86)	0.5317
ARB, n(%)	9 (36)	10(45)	0.5099
CCB, n(%)	6(24)	3(14)	0.3676
Diuretics, n(%)	5(20)	5 (23)	0.8197
Antiarrhythmic, n(%)	15 (60)	11 (50)	0.4914

Table S2. Characteristics of males with paroxysmal AF according to low (<2) and high (≥2) CHA2DS2-VASc score. The unpaired data were analyzed using parametric (unpaired t-test) and nonparametric (Mann-Whitney) t-tests. Categorized data were analyzed with the Chi-square test. A p-value < 0.05 was considered significant.

Abbreviation: BMI - body mass index; EHRA - European Heart Rhythm Association; CHA2DS2-VASc - Congestive heart failure, Hypertension, Age ≥ 75, Diabetes, Stroke, Vascular disease, Age 65–74, Sex (female); HAS-BLED - Hypertension, Abnormal renal/liver function, Stroke, Bleeding history or predisposition, Labile INR, Elderly (>65 years), Drugs/alcohol concomitantly; EF – ejection fraction; TnT - cardiac troponin T; CK-MB – creatine kinase MB, specific for the heart muscle; SBP - systolic blood pressure; DBP- diastolic blood pressure; ST-2 – suppression of tumorigenicity 2 (potential biomarker associated with cardiac stress and fibrosis); TSH – thyroid stimulating hormone; GFR, glomerular filtration rate; sCD40L – soluble ligand CD40 (release from activated platelets); t-PA – tissue plasminogen activator; PAI-1 – plasminogen activator inhibitor 1; sVCAM-1 – soluble vascular cell adhesion molecule 1; sICAM-1- soluble intercellular adhesion molecule; vWF - von Willebrand

factor; sTM – soluble thrombomodulin; ACE - angiotensin-converting enzyme inhibitor; VKA, vitamin K antagonist; NOAC, non-vitamin K antagonist oral anticoagulants; ARB - angiotensin II receptor blocker

**Parameters**	**Males CHA2DS2-VASC < 2 n=25**	**Males CHA2DS2-VASC ≥2 n=23**	**p**
Age, years	60 (48-69)	66 (54-76)	0.0018
BMI, kg/m^2	30 (22-37)	30 (22-36)	0.9400
BMI>25≤45	22 (88%)	21 (91%)	0.7081
Il-6, pg/mL	3.2 (1.9-6.7)	3.2 (2.2-11.5)	0.4956
EHRA 1	0	0	------
EHRA 2	15 (60)	10(43)	0.2524
EHRA 3	9 (36)	11(48)	0.4064
EHRA 4	1 (4)	2 (9)	0.5020

Mean HAS-BLED	1 (0-1)	2 (0-3)	<0.0001
% EF	59 (50-63)	60 (40-60)	0.8893
TnT, µg/L	0.007 (0.004-0.024)	0.009 (0.005-0.022)	0.0698
CK-MB, U/L	14 (7-31)	17 (10-46)	
SBP	125 (110-140)	125 (110-140)	0.8431
DBP	80 (55-95)	80 (60-110)	0.4543
D-dimers, mg/dL	0.13 (0.03-0.66)	0.14 (0.02-0.95)	0.9609
ST2	1.4 (0.46-4.0)	1.3 (0.49-3.7)	0.8022
TSH, µU/mL	1.2 (0.22-12.3)	1.4 (0.58-3.2)	0.8661
GFR, mL/min	73 (50-135)	82 (37-113)	0.9470
sCD40L, pg/mL	512 (274-1020)	494 (189-986)	0.8742
Cholesterol, mg/dL	196 (116-269)	138 (116-211)	0.0601
Glucose, mg/dL	105 (80-138)	98 (85-135)	0.8275
Smoking, n(%)	3 (12%)	0 (0%)	0.0862
Endothelial cell markers			
t-PA, ng/mL	1.24 (0.71-2.1)	1.42 (0.77-2.0)	0.2079
PAI-1, ng/mL	17.5 (10.2-38.1)	16.6 (9.5-49.4)	0.8103
sVCAM, ng/mL	178.1 (74.3-510.4)	237.6 (102.1-690.9)	0.1619
sICAM, ng/mL	36.9 (9.3-108.1)	23.3 (9.6-320.2)	0.1541
vWF, ng/mL	1.8 (0.46-5.4)	2.7 (0.85-5.3)	0.0550
sTM, ng/mL	3.0 (1.1-4.9)	3.3 (0.94-7.9)	0.0801
Comorbidities and medications			
Dyslipidemia, n(%)	10 (40)	5 (22)	0.1727
Hypertension, n(%)	13 (52)	18 (82)	0.0574
Heart Failure, n(%)	0 (0)	1 (4)	0.2921
Thyroid Diseases, n(%)	5(20)	5 (23)	0.8822
Statins, n(%)	7 (28)	17 (74)	0.0015
ACE inhibitor, n (%)	4(16)	7 (32)	0.2346
VKA, n(%)	2 (9)	7(30)	0.0467
NOAC, n(%)	23 (92)	16 (69)	0.0467
Beta-blockers, n(%)	17 (68)	20(87)	0.1185
ARB, n(%)	7 (30)	10(43)	0.004
CCB, n(%)	3 (12)	6 (26)	0.2116
Diuretics, n(%)	4 (16)	3(13)	0.7719
Antiarrhythmic, n(%)	6(24)	7(30)	0.6163
